# DRIVING CESSATION IS ASSOCIATED WITH POORER MENTAL HEALTH

**DOI:** 10.1093/geroni/igz038.1243

**Published:** 2019-11-08

**Authors:** Arne Stinchcombe, Carlina Marchese, Shauna Fossum, Sylvain Gagnon, Gary Naglie, Mark Rapoport, Bruce Weaver, Michel Bédard

**Affiliations:** 1 Saint Paul University (Ottawa), Ottawa, Ontario, Canada; 2 Lakehead University, Thunder Bay, Ontario, Canada; 3 University of Ottawa, Ottawa, Ontario, Canada; 4 Baycrest Health Sciences/University of Toronto, Toronto, Ontario, Canada; 5 Sunnybrook Health Sciences/University of Toronto, Toronto, Ontario, Canada

## Abstract

The Canadian Longitudinal Study on Aging (CLSA) is a longitudinal health study that will follow individuals aged 45 to 85 for 20 years. At baseline, participants completed measures related to driving status and mental health outcomes (e.g., Center for Epidemiologic Studies Depression Scale; CES-D). In this study we examined the associations between driving status and mental health outcomes. In the baseline sample, 1,415 participants reported being former drivers and 44,694 reported being current drivers. A greater proportion of former drivers were female, older, and urban-dwelling. Compared to current drivers, former drivers had lower levels of social support, poorer self-rated physical health, and less community participation. After controlling for these covariates as well as age and sex, former drivers had greater odds than current drivers of being classified as depressed (OR=2.48, 95% CI=2.21-2.79), and of reporting psychological distress (OR=2.22, 95% CI=1.87-2.62). Using data from former drivers only, we also examined associations between variables that contributed to driving cessation and depression symptoms. Former drivers had greater odds of being depressed if they reported feeling nervous or intimidated behind the wheel (OR=1.77, 95% CI= 1.11 - 2.80), or if they experienced difficulties with the licensing process (OR=1.62, 95% CI=1.07 - 2.54), before they stopped driving. As a next step we will search for factors that may modify the relationship between driving status and mental health. The identification of factors that modify the impact of driving cessation on mental health is critical to the development of interventions that will support smoother transitions to non-driving.

